# The Right to Motherhood

**Published:** 1920-10-09

**Authors:** 


**THE PUBLIC WELL-BEING**—(*continued*).

### "The Right to Motherhood."

The bold evidence given by Miss Norah March before the National Birth-Rate Commission has been followed by much discussion, and the trend of thought which she set forth has been sharply attacked. Miss March—it should be made clear—was not necessarily advocating the ideas of the right to motherhood, but was describing it as a doctrine rapidly gaining ground. Personally holding the view that love should be the basis of marriage, she also holds that as a general social guide the tenet of chastity as a preparation for marriage is the ideal, recognising, of course, that there may be individual cases wherein some other decision in regard to the rule of life may be a matter of responsible choice. If marriage is delayed till late in life, chastity may be a matter of supreme difficulty to many, while those whose circumstances make marriage impossible are confronted by the problem of entire celibacy. Our social code on these matters is

**THE PUBLIC WELL-BEING—(*continued*).**

obviously undergoing a change. It is possible that the future may see some forms of extra-marital sex relationship and parenthood finding a recognised place in our social code. There are many more women than men in this country. The wider education of girls, their entry into the world of labour—in short, their general emancipation—all tend towards a liberation of natural impulses and a desire for freedom of choice.

We are disinclined to believe that there is a rapidly growing adherence to the doctrine of the right to motherhood—at any rate, not a conscious adherence. The fact that there are many more women than men is a serious social phenomenon, but surely the tendency which has been deplored in recent times is not the claim of the right to motherhood so much as the supposed avoidance of motherhood. The wider emancipation of women should have the contrary effect to that suggested by Miss March. Women's field of work is so enlarged that spinsterhood—a name which in itself is disliked by "bachelor" women of to-day—is not the empty life it used to be; and while it is possible that, in years to come, extra-marital relations will not be considered the unsocial, not to say immoral, thing, as it is to-day, parenthood in such circumstances can hardly expect to escape at least the disapproval with which it is now regarded. Apart from moral considerations—which we hold to be vitally against it—it would give no fair chance to the children, it would place motherhood in a false position, it would smash the sanctity and the fundamental value of the home, and would effect social, as well as moral, chaos. It would be better frankly to admit the principles and practice of the harem, where there is at any rate some order. The day may come when deliberate extra-marital parenthood may not involve social ostracism, but we find it difficult to believe that it will find a recognised place in our social code.

